# Protective Role of Nuclear Factor Erythroid 2-Related Factor 2 Against Respiratory Syncytial Virus and Human Metapneumovirus Infections

**DOI:** 10.3389/fimmu.2018.00854

**Published:** 2018-04-23

**Authors:** Teodora Ivanciuc, Elena Sbrana, Antonella Casola, Roberto P. Garofalo

**Affiliations:** ^1^Department of Pediatrics, University of Texas Medical Branch, Galveston, TX, United States; ^2^Department of Microbiology and Immunology, University of Texas Medical Branch, Galveston, TX, United States; ^3^Sealy Center for Vaccine Development, University of Texas Medical Branch, Galveston, TX, United States; ^4^Sealy Center for Molecular Medicine, University of Texas Medical Branch, Galveston, TX, United States

**Keywords:** respiratory syncytial virus, human metapneumovirus, nuclear factor erythroid 2 related factor 2, antioxidant enzymes, hydrogen sulfide, lung injury, airway inflammation, airway obstruction

## Abstract

The pathogenesis of respiratory syncytial virus (RSV) infections is characterized by lower airway obstruction driven at great extent by the exuberant production of inflammatory cytokines. We have previously shown that RSV infection *in vitro* and *in vivo* results in production of reactive oxygen species along with reduction in the expression of antioxidant enzymes (AOEs), which are involved in maintaining the cellular oxidant–antioxidant balance. These events were associated with the concomitant reduction in nuclear factor erythroid 2-related factor 2 (Nrf2), a key transcription factor that controls AOE expression. The objective of the current study was to establish the role of Nrf2 in shaping innate immune responses, clinical disease, airway inflammation, and viral replication in established experimental models of intranasal RSV and human metapneumovirus (hMPV) infections, by employing mice genetically deficient for the *Nrf2* gene. Compared to control wild type (WT), mice genetically deficient in Nrf2 (Nrf2 KO) developed enhanced clinical disease, airway inflammation and pathology, and significantly greater lung viral titers following experimental infection with either RSV or hMPV. In particular, compared to control mice, RSV-infected Nrf2 KO mice lost more body weight and had increased airway obstruction at time points characterized by a remarkable increase in inflammatory cytokines and airway neutrophilia. Airway levels of AOEs and enzymes that regulate synthesis of the endogenous hydrogen sulfide (H_2_S) pathway, which we showed to play an important antiviral function, were also decreased in RSV-infected Nrf2 KO compared to WT. In conclusion, these results suggest that Nrf2 is a critical regulator of innate, inflammatory, and disease-associated responses in the airways of mice infected with viruses that are members of the *Pneumoviridae* family. Importantly, the results of this study suggest that Nrf2-dependent genes, including those controlling the cellular antioxidant and H_2_S-generating enzymes and cytokines can affect several aspects of the antiviral response, such as airway neutrophilia, clinical disease, airway obstruction, and viral replication.

## Introduction

Acute bronchiolitis is a viral lower respiratory tract infection (LRTI) that represents the primary cause of hospitalization for children in the first year of life. The orthopneumovirus respiratory syncytial virus (RSV), a member of the *Pneumoviridae* family is the causative agent in more than 50% of the cases. In the United States, 75,000–125,000 hospitalizations related to RSV occur each year in children <1 year of age, and RSV infection results in approximately 1.5 million outpatient visits among children <5 years of age (1, 2). Given the fact that RSV infections do not provide permanent immunity, reinfections can reoccur throughout life and cause serious respiratory disease in certain adult populations, including frail elderly and subjects with chronic heart and lung diseases (3). There are no vaccines or specific antiviral therapies currently licensed to prevent or treat RSV infections. A significant obstacle to the development of therapeutic strategies is our still limited understanding of the pathogenic mechanisms that determine the severity of LRTI caused by RSV. Although infants with certain risk factors (prematurity, chronic lung disease, congenital heart disease, or immunodeficiencies) have an increased risk for more severe RSV disease, the large majority of infants with RSV infections that require hospitalization were previously healthy (4, 5). Therefore, the spectrum of RSV disease severity in otherwise previously healthy infants, points to both host determinants and virus-specific factors that determine the outcome of infection (4). Lung inflammation and airway obstruction, driven by an exuberant innate mucosal immune response and cytokine production is recognized to play a central pathogenic role (6, 7). Excess neutrophils are found in the airways of infants with acute RSV bronchiolitis as well as in the bronchoalveolar lavage (BAL) fluid of RSV-infected children (8, 9). At the same time, infants with greater viral quantities in the respiratory tract secretions are at greater risk for prolonged hospitalization, intensive care unit stay, and mechanical ventilation (10), possibly by causing direct lung injury.

Reactive oxygen species, such as the superoxide radical anion $\left({\text{O}}_{2}^{-•}\right)$ and hydrogen peroxide (H_2_O_2_), have been directly implicated in tissue damage, inflammatory disorders, pulmonary disease, and infections [reviewed in Ref. (4, 11)]. We have previously shown that RSV is a potent inducer of reactive oxygen species (ROS) in epithelial cells and in the airways but, contrary to what has been shown in certain viral infections, they do not contribute to the host anti-RSV response; rather they cause lung inflammation and clinical disease (12). In addition, we have shown that ROS function as intermediate signal in the transcriptional regulation of cytokine and chemokine genes and cause oxidative damage to the airways (12–14). Indeed, treatment of RSV-infected mice with antioxidant molecules reduces inflammation, clinical disease, and airway hyperresponsiveness (AHR) (12). Our most recent study shows that RSV infection in epithelial cells (15), in experimentally infected mice and in naturally infected children (16) causes a significant decrease in the expression of most antioxidant enzymes (AOEs) and as result it disrupts the pro-oxidant–antioxidant balance (4).

Transcription of many oxidative-stress-inducible genes is regulated in part through *cis*-acting antioxidant responsive element (ARE) sequences. This element has been identified in the regulatory regions of genes encoding detoxification enzymes, such as NQO1 (NADPH:quinone oxidoreductase), as well as many AOEs, including Cu/Zn-superoxide dismutase (SOD1), catalase, heme oxygenase 1, glutathione S-transferase, and glutathione-generating enzymes, such as glutamate cysteine ligase (GCLC) [reviewed in Ref. (17)]. NF-E2-related factor 2 (Nrf2) is an important redox-responsive protein that helps to protect cells from oxidative stress and injury. It is a basic leucine zipper transcription factor that is normally bound in the cytosol to a cytoskeleton-associated inhibitor called Kelch-like ECH-associated protein 1 (Keap1). Electrophile-induced release of Nrf2 is proposed to involve covalent modifications of Keap1 and/or Nrf2 in the cytoplasm. Such modifications include oxidation of key cysteine residues in Keap1, phosphorylation of Nrf2, and switching of Cullin-3-dependent ubiquitination from Nrf2 to Keap1, leading to the degradation of Keap1, and the stabilization and activation of Nrf2 (18). The released Nrf2 then translocates to the nucleus and binds to ARE sites to promote gene transcription (17). Cho et al. using Nrf2-deficient mice have shown that the Nrf2-ARE pathway plays a key role in antiviral host response to RSV (19) and we have shown in previous studies that decrease in AOEs expression in epithelial cells as well as in the lungs of mice infected with RSV was associated with a significant decrease in nuclear expression of Nrf2 (15, 16). Furthermore, children with naturally acquired RSV infections had significant increase in markers of oxidative injury and a significant decrease in AOE expression in their nasopharyngeal secretions (NPS), which correlated with the severity of clinical illness (16), suggesting that RSV-induced oxidative damage *in vivo* is the result of an imbalance between ROS production and airway antioxidant defenses. Therefore, the current study was designed to test the role of Nrf2 in shaping innate immune responses, clinical disease, airway inflammation, and viral replication in an established experimental model of RSV infection, by employing adult/aged mice that were genetically deficient in the *Nrf2* gene.

## Materials and Methods

### RSV and Human Metapneumovirus (hMPV) Preparations

Respiratory syncytial virus long strain was grown in HEp-2 cells (American Type Culture Collection, Manassas, VA, USA) and purified by polyethylene glycol precipitation, followed by centrifugation on 35–65% discontinuous sucrose gradients as described elsewhere (20, 21). The virus titer of the purified RSV was determined by a methylcellulose plaque assay and ranged from 8–9 log10 plaque forming units (PFU)/ml. The hMPV strain CAN97-83 was obtained from the Centers for Disease Control, Atlanta, GA, USA, with permission from Guy Boivin at the Research Center in Infectious Diseases, Regional Virology Laboratory, Laval University, Quebec, Canada. Virus was propagated in LLC-MK2 cells (American Type Culture Collection) in minimal essential medium (without serum) containing 1 µg trypsin/ml, followed by purification on a 60% sucrose cushion (22). Virus titer was determined by a cell-based immunoassay. For that, sucrose-purified virus was serially diluted (log10) on LLC-MK2 cell monolayers in 96-well flat-bottom plates. Forty-eight hours later, monolayers were washed and incubated with guinea pig anti-hMPV antibody (MedImmune, Inc., Gaithersburg, MD, USA) and stained with horseradish peroxidase-labeled anti-guinea pig antibody (Zymed, San Francisco, CA, USA). Infected cells were detected using 3-amino-9-ethyl-carbazole, infectious units being enumerated by light microscopy. The final viral titer was expressed as PFU/ml. Virus pools are aliquoted, quick-frozen in liquid nitrogen, and stored at −70°C until used. Viral preparations are routinely tested for LPS and cytokine contamination.

#### Neutrophil-Specific Probe

Neutrophil-Specific, near infrared (NIR) Fluorescent Imaging Agent (cFLFLF-PEG_76_-Cyanine7) was purchased from Kerafast (Boston, MA, USA). The neutrophil probe was freshly prepared in PBS prior to use in mice according to the manufacturer instructions.

#### Ethics Statement

All procedures involving mice in this study were performed in accordance with the recommendations in the Guide for the Care and Use of Laboratory Animals of the National Institutes of Health. The protocol was approved by the Institutional Animal Care and Use Committee of the University of Texas Medical Branch at Galveston.

#### Mice and Infection Protocol

The study has been approved by the UTMB IACUC (protocol number 9001002 and 0808049). Heterozygous Nrf2^+/−^ mice (on C57BL6 background) were purchased from Jackson Laboratory (Bar Harbor, ME, USA). In this Nrf2^−/−^ strain, exons 4 and 5 of the mouse *Nfe2l2* gene, which encodes the basic leucin zipper domain that controls transcriptional activation, has been replaced by a LacZ reporter gene. Heterozygous mice were crossbred to generate homozygous Nrf2 ^−/−^ (Nrf2 KO) mice as well as wild type littermates controls [Nrf2 ^+/+^, wild type (WT)]. Genotyping for Nrf2 status was performed by PCR amplification of genomic DNA. Pathogen-free breeding colonies of Nrf2^+/−^, Nrf2 ^−/−^, and Nrf2 ^+/+^ were maintained at the University of Texas Medical Branch at Galveston and experiments were performed using 12- to 15-month-old Nrf2 KO mice and WT age-matched littermates of both genders. Under light anesthesia, were infected intranasally (i.n.) with 50 µl of RSV diluted in PBS at a dose of 10^7^ PFU or mock inoculated using the same volume of control buffer. Daily determinations of body weight and illness score, collection of BAL for differential cell counts and cytokines, chemokines, and type I interferons (IFNs) measurements, lung neutrophil counts by flow cytometry, lung viral titration, nuclear translocation of NF-κB/RelA in the lungs, pulmonary histopathology, and pulmonary function testing were performed as previously described (12, 23–25). Antioxidant (SOD1 and catalase) and hydrogen sulfide (H_2_S)-generating enzyme (CSE, CBS, and 3MST) mRNA expression in lung tissue was analyzed by quantitative real-time PCR (qRT-PCR) (24, 26). In some experiments Nrf2 KO and WT mice, were infected i.n. with 5 × 10^6^ PFU of human metapneumovirus (hMPV, CAN97-83 strain) for assessment of disease parameters, BAL differential cell count, cytokines and chemokines, and pulmonary function testing (27, 28).

### Clinical Disease

Animals from all groups were evaluated on a daily basis for weight loss, illness score, and presence of any respiratory symptoms. The percentage of body weight change was plotted over time. A clinical illness score for mice was used to measure the severity of clinical disease (0—healthy; 1—barely ruffled fur; 2—ruffled fur but active; 3—ruffled fur and inactive; 4—ruffled fur, inactive, and hunched; and 5—dead). These parameters have been shown to closely correlate with lung pathology in experimental infection of mice (12, 24, 25, 27).

### Broncholaveolar Lavage

Broncholaveolar lavage was collected *via* the trachea by flushing the lungs twice with 1 ml of ice-cold PBS. A total of 100 µl of BAL fluid was used for cytospin analysis, and the rest was immediately centrifuged and stored at −80°C. Total number of BAL cells was counted with a hemacytometer and viability was assessed by trypan blue. BAL differential cell counts were determined using morphogenic criteria under light microscopy of Protocol HEMA3 (Fisher Scientific) stained cytospins with a total count of 200 cells per slide.

### Measurement of Cytokines, Chemokines, and IFNs

Levels of cytokines and chemokines in BAL fluid were determined with the Bio-Plex Pro Mouse Group I 23-plex panel (Bio-Rad Laboratories, Hercules, CA, USA). The lower limit of detection for all cytokines measured by this assay is 3 pg/ml. IFN-α and IFN-β were measured by commercial enzyme-linked immunosorbent assays (ELISA), following the manufacturer’s protocol (PBL Biomedical Laboratories, Piscataway, NJ, USA). The range of sensitivity of the assay is 12.5–400 pg/ml for IFN-α, and 12.5–1,000 pg/ml for IFN-β.

### RSV Titration of Lung Tissue

Lungs were removed from infected animals at day 5 after RSV infection. Tissue samples were homogenized in 1 ml of Dulbecco’s modified Eagle’s medium and centrifuged twice at 14,000 rpm for 1 min at 4^o^C. Serial twofold dilutions of the supernatant were determined by plaque assay on HEp-2 cells under methylcellulose overlay. Plaques were visualized 5 days later, and virus titers will be expressed as log10 PFU/gram tissue.

### Pulmonary Histopathology

Mice were euthanized at days 5 and 7 post-infection, and the entire lung was perfused, removed, and fixed in 10% buffered formalin following by paraffin embedding. Multiple 4-µm longitudinal cross-sections were stained with hematoxylin and eosin (H&E). The slides were analyzed and scored for cellular inflammation under light microscopy by a board-certified pathologist with expertise in mouse lung, unaware of the infection status of the animals. Two separate grading systems were used to assess the lung inflammation. The first grading system, adapted from Ref. (23), measured the percentage of abnormal perivascular spaces in the tissue sections. The second grading system assigned a 0–4 grade based on severity (0 = normal, 4 = severe pathologic changes) to four different parameters: perivasculitis, bronchiolitis, alveolitis, and necrosis. Ten high power fields were examined for each slide, and average grades were compared between groups and analyzed to determine whether observed differences were statistically significant.

### Pulmonary Function

Airway hyperresponsiveness was assessed in unrestrained mice at different times after infection using whole-body barometric plethysmography (Buxco, Troy, NY, USA) to record enhanced pause (Penh), as previously described (12, 24). Penh is a dimensionless value that represents a function of the ratio of peak expiratory flow to peak inspiratory flow and a function of the timing of expiration. Respiratory activity was recorded for 5 min, to establish baseline Penh values. Mice were subsequently exposed to increasing doses of nebulized methacholine (3.25, 6.25, 12.5, 25, and 50 mg/ml) for 2 min, and data were recorded for another 3 min.

### Flow Cytometry of Lung Cells

For flow cytometry analysis, lungs were collected at day 1 after RSV or mock infection and digested with collagenase, as previously described (24, 25, 27, 28). Cells were passed through nylon mesh to get a single cell suspension, and incubated with anti-FcγRIII/FcγRII mAb (anti-mouse CD16/CD32; BD Biosciences, San Diego, CA, USA) to reduce nonspecific binding, for 30 min at 4°C. After washing, cells were stained with the following anti-mouse antibodies: anti-F4/80 APC, anti-CD11b PerCP-Cy5.5, and anti-Ly6G FITC (for neutrophils). After incubation for 30 min at 4°C with the antibodies, cells were washed and then fixed in 200 µl of 1% paraformaldehyde in PBS. All the antibodies were purchased from BD Pharmingen, San Jose, CA, USA, except anti-F4/80 which was obtained from ebioscience, San Diego, CA, USA. Corresponding isotype Abs were used as controls. Cells were acquired with a FACS Canto flow cytometer equipped with Cell Quest software (both from Becton Dickinson Immunocytometry Systems, San Jose, CA, USA). Analysis was performed by using the FlowJo Software (Tree Star, NJ, USA).

### Quantitative Real-Time PCR

Total RNA was extracted by using a ToTALLY RNA kit (catalog number AM1910; Ambion, Austin, TX, USA). RNA samples were quantified by using a NanoDrop spectrophotometer and quality was analyzed on an RNA Nano-drop by using the Agilent 2100 bioanalyzer (Agilent Technologies). Synthesis of cDNA was performed with 1 µg of total RNA in a 20 µl reaction mixture by using the TaqMan Reverse Transcription Reagents kit from ABI (catalog number N8080234; Applied Biosystems). Amplification was done using 1 µl of cDNA in a total volume of 25 µl using the Faststart Universal SYBR green Master Mix (Roche Applied Science #04913850001). Viral genome copy numbers and RSV N gene copy numbers (primer sequences and conditions) were measured as previously described (24, 26). The mRNA sequences for SOD1, catalase, CSE, CBS, and 3-mercaptopyruvate sulfurtransferase (3-MST) reported under GenBank accession numbers X06683.1, L25069, NM_145953, NM_144855.3, and NM_138670 were used to design amplification primers for qRT-PCR assay (24). 18S RNA was used as housekeeping gene for normalization. PCR assays were run in the ABI Prism 7500 Sequence Detection System. Triplicate cycle threshold (*C*_T_) values were analyzed in Microsoft Excel by the comparative *C*_T_ (ΔΔ*C*_T_) method according to the manufacturer’s instructions (Applied Biosystems). The amount of target $\left(2^{-\Delta \Delta C_{\text{T}}}\right)$ was obtained by normalization to the endogenous reference (18S) sample. RNA isolation, primer design, and qRT-PCR assays were performed at Molecular Genomic Core, UTMB, Galveston.

### Western Blotting

Nuclear extracts of uninfected and infected lungs were prepared as previously described (16, 23). Equal amount of proteins (20 µg per sample) were then boiled in 2× Laemmli buffer and resolved on SDS-PAGE gels. Proteins were transferred onto a polyvinylidene difluoride membrane (Amersham, Piscataway, NJ, USA), and nonspecific binding sites were blocked by immersing the membrane in Tris-buffered saline–Tween (TBST) containing 5% skim milk powder, overnight at 4°C. After a short wash in TBST, membranes were incubated with the primary antibody NF-κB p65 diluted 1:1,000 overnight at 4°C, followed by incubation with horseradish peroxidase (HRP)-conjugated secondary antibody (Santa Cruz, CA, USA), diluted 1:5,000 in TBST, for 1 h at room temperature. Finally, after washing three times with TBST, proteins were detected by using an enhanced chemiluminescence system (RPN 2016; Amersham, GE Healthcare, United Kingdom). Membranes were stripped and reprobed with anti-β-actin antibody for loading control. Densitometric analysis of band intensities was performed using UVP VisionWorksLS Image Acquisition and Analysis Software 8.0 RC 1.2 (UVP, Upland, CA, USA). Antibodies used for Western blot assays were NF-κB p65 rabbit mAB (C2284) and anti-β-actin (4967) from Cell Signaling, Technology, MA.

### *In Vivo* Imaging of Neutrophils

Real-time visualization of neutrophils in the lung of infected mice was performed using the NIR Fluorescent Imaging Agent (29). One hour prior to imaging, 2 nmol of the neutrophil-specific, NIR Fluorescent Imaging Agent was administered to Nrf2 KO and WT RSV-infected mice and mock controls by way of tail vein injection or intratracheal instillation. Lungs were excised and imaged *ex vivo* using an IVIS 200 Spectrum (Perkin-Elmer, Waltham, MA, USA) and analyzed using Living Image software (Perkin-Elmer, Waltham, MA, USA). Images were collected after 1 s of exposure utilizing a 745 nm excitation and 800 nm emission filters. To depict the differences in intensity of the signal, fluorescence are represented in the images with a pseudocolor scale ranging from yellow (most intense) to dark red (least intense).

### Statistical Analysis

The data were analyzed by one-way ANOVA followed by Tukey’s *post hoc* test for samples with unequal variances (GraphPad Prism 5.02; GraphPad Software, Inc., San Diego, CA, USA). Results are expressed as mean ± SEM for each experimental group unless otherwise stated and *p* < 0.05 value was selected to indicate significance.

## Results

### Nrf2 Deficiency Exacerbates Disease Severity and Airway Dysfunction in RSV Infection

**Figure 1:**
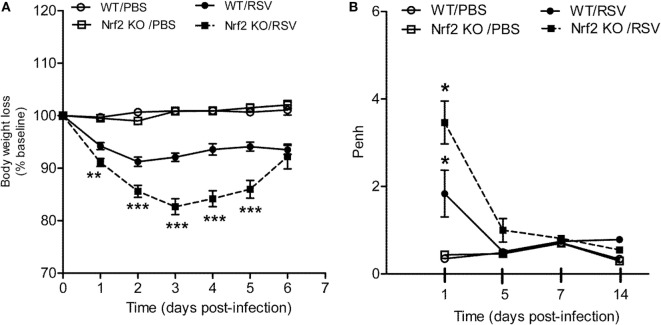
NF-E2-related factor 2 (Nrf2) gene deficiency in mice exacerbates clinical disease and airway obstruction following respiratory syncytial virus (RSV) infection. Under light anesthesia, groups of Nrf2 ^−/−^ (KO) and Nrf2 ^+/+^ [wild-type (WT) control] mice were infected intranasally (i.n.) with 10^7^ plaque-forming units (PFU) of RSV or PBS. **(A)** Mice were monitored daily for changes in body weight and disease manifestation. Data are expressed as mean ± SEM (*n* = 4–12 mice/group). **p < 0.01, ***p < 0.001 when compared with WT/RSV at days 1, 2, 3, 4, and 5 post-infection. **(B)** Airway obstruction represented by baseline Penh was assessed by unrestrained plethysmography (Buxco Electronics, Inc., Sharon, CT). RSV-infected Nrf2 KO mice have increased airway obstruction compared to RSV-infected WT mice. Data are expressed as mean ± SEM (*n* = 3–8 mice/group). *p < 0.05 WT/RSV compared with WT/PBS, Nrf2 KO/RSV compared with Nrf2 KO/PBS, and Nrf2 KO/RSV compared with WT/RSV at day 1 post-infection.

To understand the role of Nrf2 in the context of RSV infection, Nrf2 KO and WT control mice were infected with RSV (10^7^ PFU/mouse) and monitored daily for clinical disease (i.e., body weight loss). Mock-infected mice did not display any signs of sickness or weight loss over the 6-day monitoring period, indicating that Nrf2 deficiency alone does not lead to clinical illness in mice. On the other hand, in RSV-infected mice, Nrf2 deficiency resulted in an enhanced disease severity compared with WT mice (Figure 1A). Nrf2 KO RSV-infected mice exhibited significant more body weight loss (~17%) at peak of clinical disease (day 3) than WT mice (8%, with disease peaking at day 2 post-infection). In addition, recovery from weight loss at days 4 and 5 post-infection was delayed in Nrf2 KO mice when compared with WT controls. Similar body weight loss was observed in both groups at day 6 post-infection. RSV-infected mice showed ruffle fur, but no significant difference in total illness score was observed between WT and Nrf2 KO animals (data not shown).

Next, to assess the effect of Nrf2 deficiency on pulmonary function after RSV infection airway obstruction and AHR in response to methacholine challenges were assessed by whole-body plethysmography (Buxco Electronics, Inc., Sharon, CT, USA) and expressed as enhanced pause (Penh). Nrf2 deficiency did not alter baseline Penh values or AHR to methacholine in mock-infected animals when compared with WT control mice. However, as shown in Figure 1B, RSV-infected WT mice developed significant airway obstruction compared with mock controls animals at day 1 post-infection (*p* < 0.05), and returning to baseline by day 5 post-infection and later time points tested (days 7 and 14). In addition, Nrf2 KO mice had significantly more airway obstruction compared to WT RSV-infected mice starting at day 1 post-infection (*p* < 0.05). In the case of AHR, RSV-infected mice showed dose-dependent increase in airway AHR in response to aerosolized methacholine at day 5 post-infection compared to mock-inoculated mice, but there are no significant differences between Nrf2 KO and WT mice (data not shown). Similarly, no differences in AHR between Nrf2 KO and WT groups were observed at later time points of infection (days 7 and 14).

### Increased Viral Replication in Nrf2-Deficient Mice

**Figure 2:**
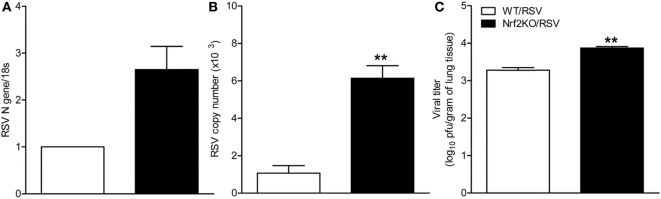
NF-E2-related factor 2 (Nrf2) KO mice have increased viral replication in the lung. Nrf2 KO and wild type (WT) mice were infected intranasally with 10^7^ plaque-forming units of respiratory syncytial virus (RSV) or PBS. Lungs were excised and viral replication was determined by quantitative real-time PCR (RT-PCR) and by plaque assay. At day 1 post-infection RSV N gene **(A)** and RSV genome copy number **(B)** in the lung were determined by RT-PCR. **(C)** Viral titration was performed at day 5 post-infection by a methylcellulose plaque assay. Data are expressed as mean ± SEM (*n* = 3–4 mice/group). **p < 0.01 when compared with WT/RSV at days 1 and 5 post-infection.

To determine whether Nrf2 deficiency would alter replication of RSV in the lung, Nrf2 KO and WT mice were sacrificed at days 1 and 5 after infection and lung tissue was collected to determine virus replication and titer by qRT-PCR and by plaque assay. Compared to WT mice, significantly higher RSV N gene and genome copies number were found in the lungs of Nrf2 KO RSV-infected mice at day 1 post-infection (Figures 2A,B). Moreover, RSV peak titers (day 5) were significantly greater in the lungs of Nrf2 KO mice compared to WT mice (*p* < 0.01) (Figure 2C).

### Nrf2 Deficiency Exacerbates Pulmonary Inflammation in RSV-Infected Mice

**Figure 3:**
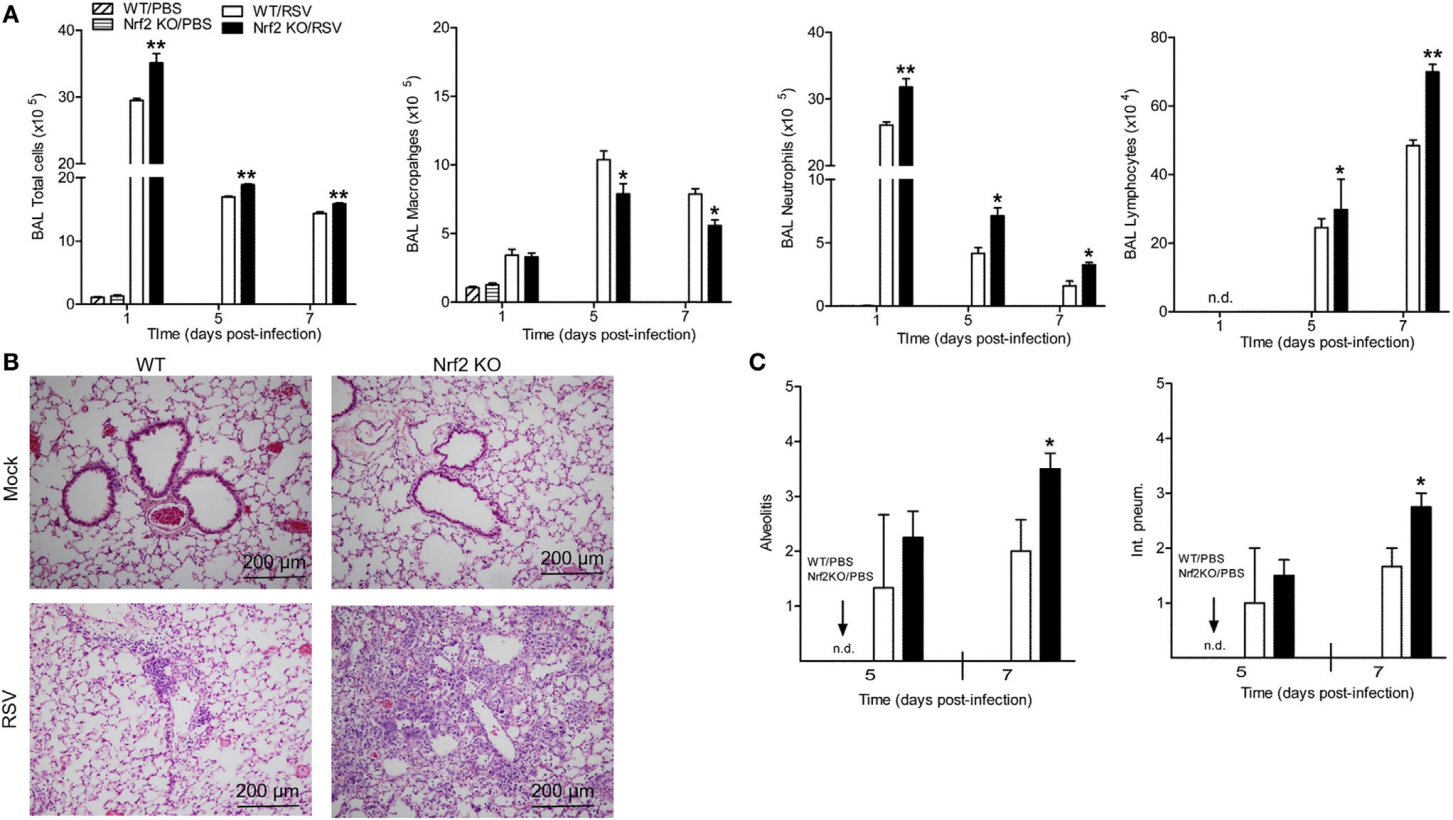
NF-E2-related factor 2 (Nrf2) gene deficiency increases airway neutrophilia and lung inflammation in respiratory syncytial virus (RSV)-infected mice. Nrf2 KO and Nrf2 wild type (WT) mice were infected with RSV or PBS. **(A)** Bronchoalveolar lavage was collected at different time points after infection for differential cell counts by Protocol HEMA3-stained cytospins. **(B)** Lung samples were harvested at days 5 and 7 post-infection, formalin-fixed for slide preparation, and hematoxylin and eosin stained. Representative stained lung tissue sections from the indicated treatment at day 7 post-infection. **(C)** Alveolitis and interstitial pneumonitis scored on lung sections. The bar graph represents mean ± SEM (*n* = 2–4 mice/group). *p < 0.05, **p < 0.01 when compared with WT/RSV mice.

Next, we investigated whether Nrf2 could be involved in RSV-mediated inflammatory response in the lung. Mock or RSV-infected Nrf2 KO and WT mice were sacrificed at different days post-infection to collect BAL samples for total and differential cell count. The total number of cells was significantly greater in BAL samples of Nrf2 KO compared to WT at 1, 5, and 7 days post-infection (Figure 3A). The increase in total BAL cells was primarily due to the increase in the number of neutrophils and lymphocytes in Nrf2 KO mice, which had concomitantly less macrophages compared to WT littermates (macrophages are mostly observed in uninfected BAL samples). No eosinophils were observed in BAL samples of either group. Furthermore, the effect of Nrf2 on pulmonary inflammation was confirmed by the pathology analysis. For histopathology studies, lungs from mock- and RSV-infected Nrf2 KO and WT mice were collected at days 5 and 7 after infection, formalin-fixed, H&E stained, and qualitatively and quantitatively analyzed using an established grading score (from 0, absent to 4, severe). Representative lung specimens for each group at day 7 after infection are shown in Figure 3B. Mock-infected animals from both groups had no inflammatory infiltrates in the lung (Figure 3B, upper panels). Lungs from RSV-infected WT mice showed mild perivascular infiltrates, mostly consisting of mononuclear cells (Figure 3B, left lower panel). On the other hand, lungs from RSV-infected Nrf2 KO mice showed diffuse inflammation with perivasculitis, peribronchiolitis, alveolitis, and vasculitis. Overall, quantification of the pulmonary inflammation by alveolitis and interstitial pneumonitis scores indicated significantly greater airway pathology at day 7 in RSV-infected Nrf2 KO mice compared to WT (*p* < 0.05, Figure 3C).

### RSV Exacerbates Lung Neutrophilia in Nrf2-Deficient Mice

**Figure 4:**
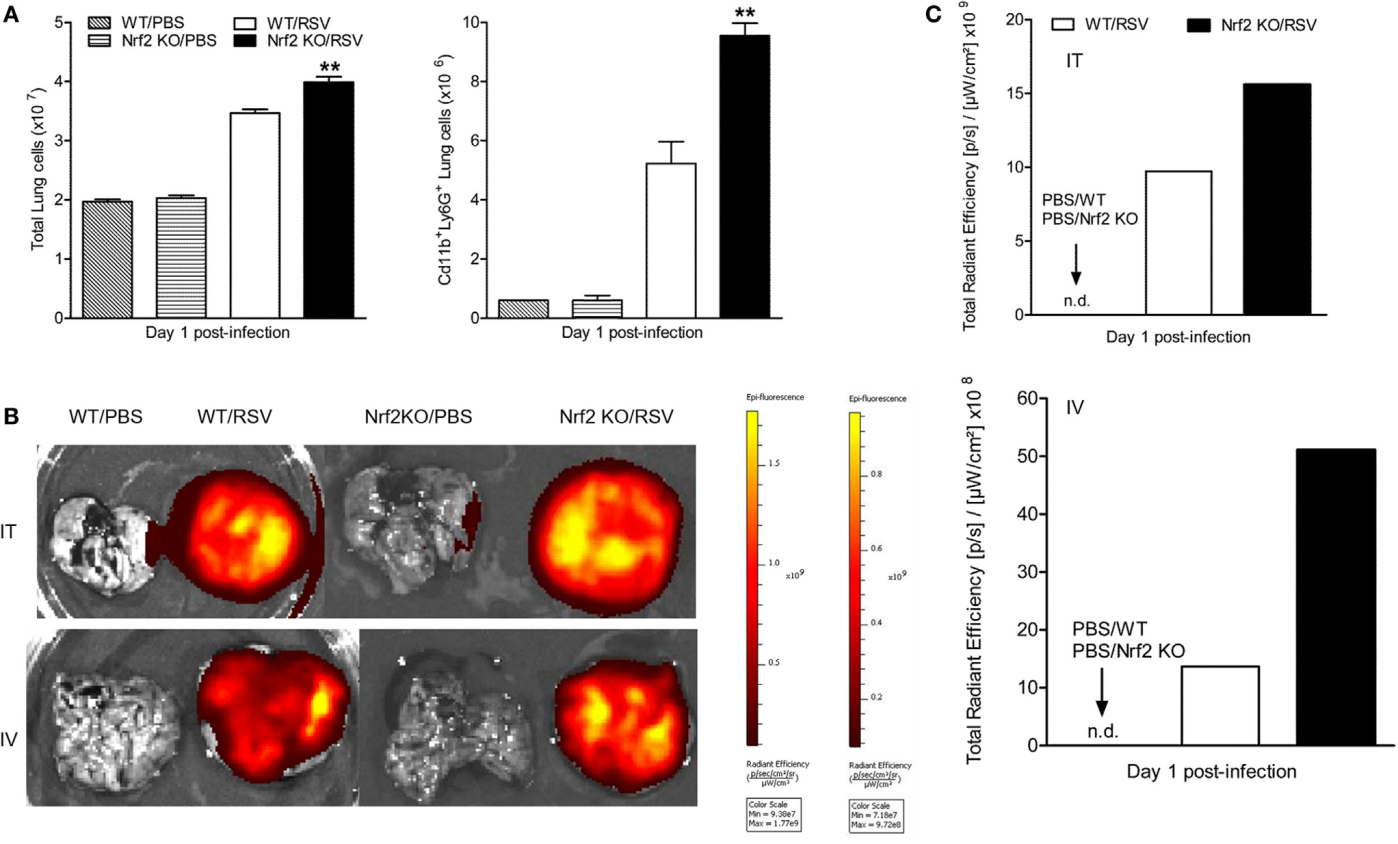
NF-E2-related factor 2 (Nrf2) deficiency leads to increased neutrophil infiltration in the lungs in response to respiratory syncytial virus (RSV) infection. Nrf2 KO and wild typr (WT) mice were infected with RSV or PBS and lungs collected at day 1 post-infection. **(A)** Neutrophils (CD11b^+^Ly6G^+^) recruitment to the lung by flow cytometry analysis. Imaging **(B)** and quantification **(C)** of lung neutrophils using the near infrared fluorescent imaging agent by intratracheal (i.t., upper panels) or intravenous tail injection (i.v., lower panels). Fluorescence intensity is represented in the images with a pseudocolor scale ranging from yellow (most intense) to dark red (least intense) and quantified in specific total radiant efficiency per lung region of efficiency, using the Living Image software. The bar graph represents mean ± SEM (*n* = 2–4 mice/group). **p < 0.01 when compared with WT/RSV mice.

To further analyze the contribution of Nrf2 in the process of neutrophil recruitment to the lung observed at early time points after RSV infection, we conducted additional studies by flow cytometry. For that, lungs were harvested at day 1 post-infection. Lung single-cell suspensions were prepared from Nrf2 KO and WT mock- and RSV-infected mice and neutrophils were gated as CD11b^+^/Ly6G^+^ cells. Following RSV inoculation, Nrf2 KO mice had significantly higher total cell numbers (mostly neutrophils) compared with WT mice (*p* < 0.01) (Figure 4A). Neutrophil influx was significantly greater in Nrf2 KO mice (9.55 ± 0.428 × 10^6^) compared to WT (5.22 ± 0.746 × 10^6^). Next, to monitor neutrophil trafficking *in vivo* in real time we used the neutrophil-specific, fluorescent imaging agent, NIR. This reagent is a Cyanine7-conjugate, PEG-modified hexapeptide that specifically binds the formylpeptide receptor of neutrophils. One hour prior to imaging, NIR was administered to mock- and RSV-infected animals, either intratracheally or intravenously, and 24 h later animals were euthanized and their lungs were excised and imaged. As shown in Figure 4B, no fluorescence signal was detected in mock-infected Nrf2 KO or WT mice by either mode of administration of the NIR agent. On the other hand, in RSV-infected mice, the fluorescence signal was well detectable in the lungs and greater in Nrf2 KO animals compared to WT, as shown in Figure 4C (fluorescence intensity signal detected using the intratracheal administration of neutrophil-specific agent). Taken together, these studies provide strong evidence that RSV-induced neutrophil migration to the lung is greatly enhanced in the absence of Nrf2 gene.

### Nrf2 Deficiency Increases the Release of Lung Cytokines and Chemokines After RSV Infection

**Figure 5:**
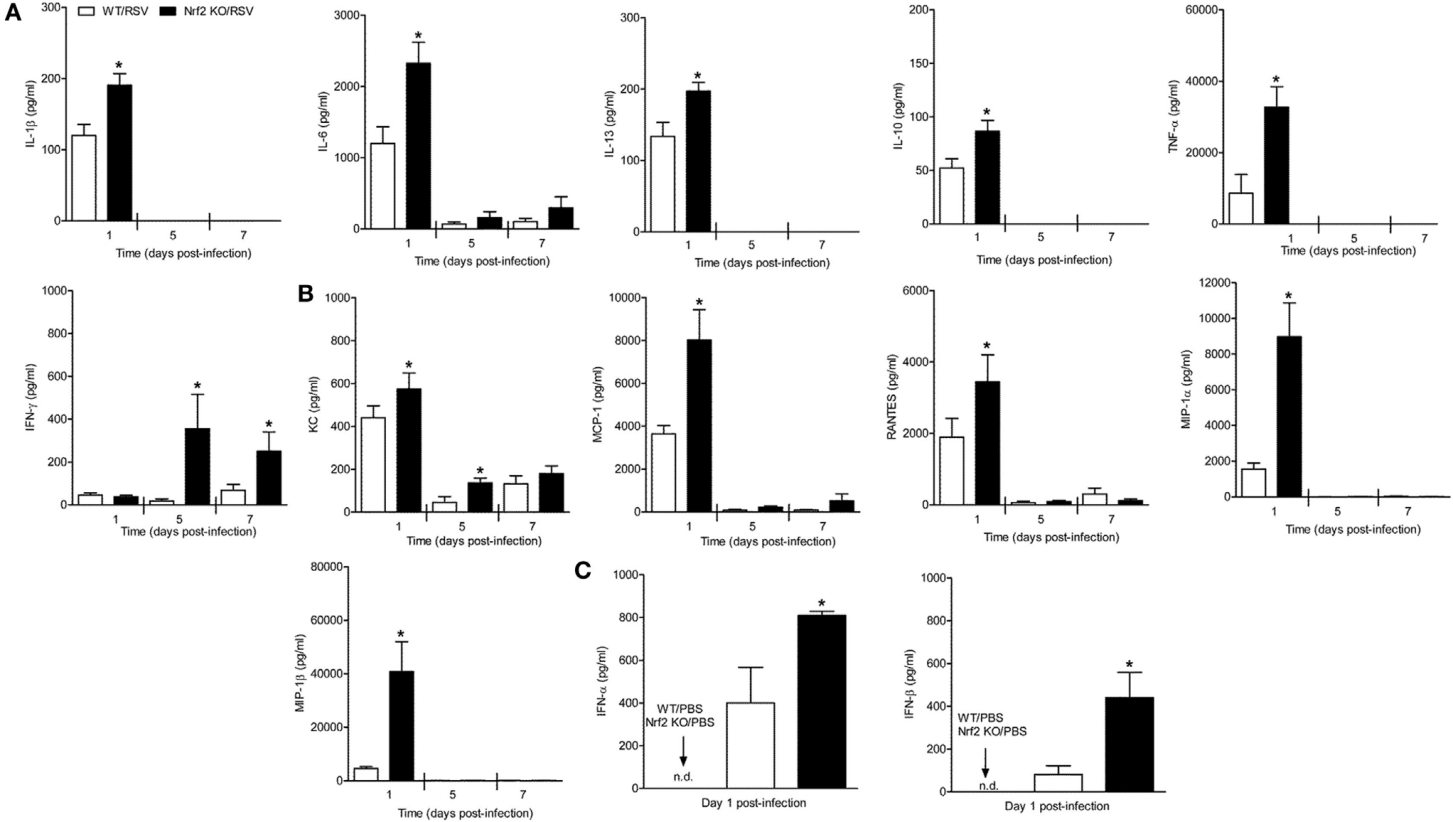
NF-E2-related factor 2 (Nrf2) gene deficiency is associated with increased secretion of cytokines chemokines and type I interferon (IFN) in response to respiratory syncytial virus (RSV) infection. Nrf2 KO and Nrf2 wild type (WT) mice were infected with RSV or PBS. The bronchoalveolar lavage samples were collected at different time points after infection and analyzed for cytokines **(A)** and chemokines **(B)** by Bio-Plex, and for type I IFN by enzyme-linked immunosorbent assays **(C)**. The bar graph represents mean ± SEM (*n* = 3–6 mice/group). *p < 0.05 when compared with WT/RSV.

Respiratory syncytial virus is a potent inducer of cytokines and chemokines, which have been shown to play an important immunoregulatory role and contribute to lung inflammation and disease severity. In our mouse model, the peak of chemokine production occurs during the first 2 days of infection (30). Thus, to investigate the role of Nrf2 in the regulation of RSV-induced inflammatory response, the concentration of cytokines and chemokines was measured in Nrf2 KO mice and compared with WT littermates. Mice were infected with PBS or RSV and at days 1, 5, and 7 after infection, BAL samples were collected from each group of mice and assessed for cytokines by a multi-plex cytokine array. Concentrations of inflammatory and immunomodulatory cytokines IL-1β, IL-6, TNF-α, IL-10, and IL-13 were significantly greater in BAL samples of Nrf2 KO mice compared to WT controls at day 1 after infection (*p* < 0.05) (Figure 5A). Also, we found strikingly increased levels of BAL IFN-γ at days 5 and 7 post-infection in Nrf2 KO mice compared to WT controls (*p* < 0.05). Similar results were observed with regards to chemokines, including CCL5 (RANTES), CCL3 (MIP-1α), CCL4 (MIP-1β), CCL2 (MCP-1), and CXCL1 (KC) (Figure 5B). Concentrations of type I IFN-α and -β were also significantly greater in BAL samples of Nrf2 KO mice compared to WT controls (Figure 5C).

### RSV Induced Greater NF-κB Activation in Lungs of Nrf2-Deficient Mice

**Figure 6:**
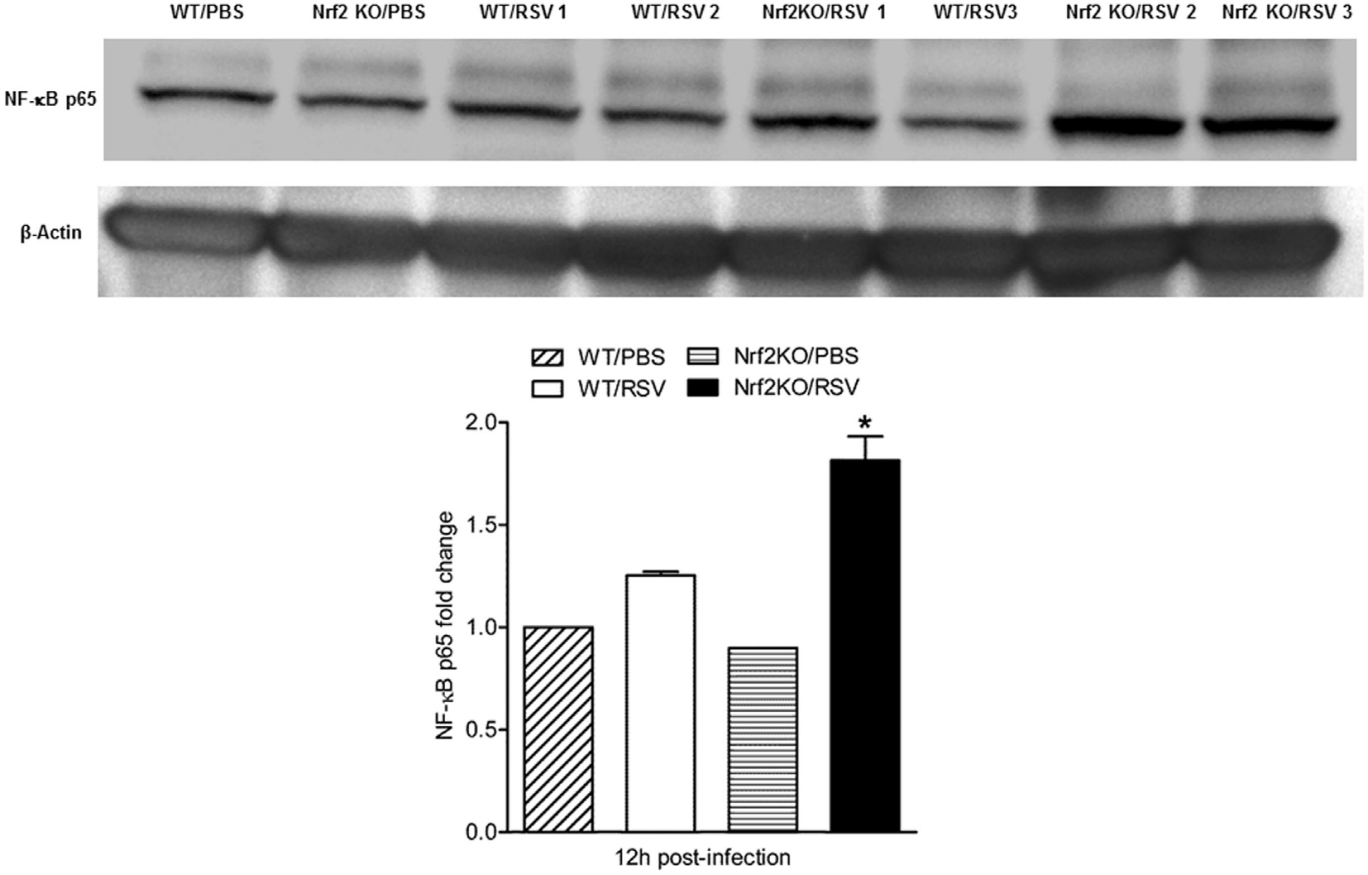
NF-E2-related factor 2 (Nrf2)-deficient mice have enhanced NF-κB nuclear translocation in the lung. Nrf2 KO and wild type (WT) mice were infected with respiratory syncytial virus (RSV) or PBS inoculated for 12 h. Nuclear proteins were isolated from lungs and subjected to Western blot analysis with anti-NF-κB p65 antibody. For loading controls, membranes were stripped and reprobed with anti-β actin Ab. Densitometric analysis of NF-κB p65 band intensity is shown after normalization to β-actin. The groups were analyzed by one-way ANOVA followed by Tukey’s *post hoc* test. Data are shown as mean ± SEM. *p < 0.05 relative to WT/RSV-infected mice.

The transcription factor nuclear factor (NF)-κB plays a major role in innate inflammation by controlling the expression of cytokines, inducible chemokines, as well as mucosal IFNs (23, 31, 32). We have previously shown, using a BALB/c mouse model, that RSV potently and specifically activates NF-κB *in vivo* and plays a central role in regulating RSV-induced disease and pathology (23, 33). Thus, we assessed NF-κB activation in RSV-infected mice lacking Nrf-2, as a possible mechanism underlying our findings of enhanced cytokines and inflammation in KO mice. Nuclear proteins were isolated from the lungs of Nrf2 KO and WT mice (23, 33, 34), that were either PBS inoculated or infected with RSV for 12 h were subjected to Western blot analysis with anti-NF-κB p65 antibody. The results of these experiments demonstrated a significantly greater abundance of lung nuclear p65 in RSV-infected Nrf2 KO compared to WT mice (Figure 6, *p* < 0.05).

### Expression of AOEs and H_2_S-Generating Enzymes Are Reduced in Nrf2 KO Mice in Response to RSV Infection

**Figure 7:**
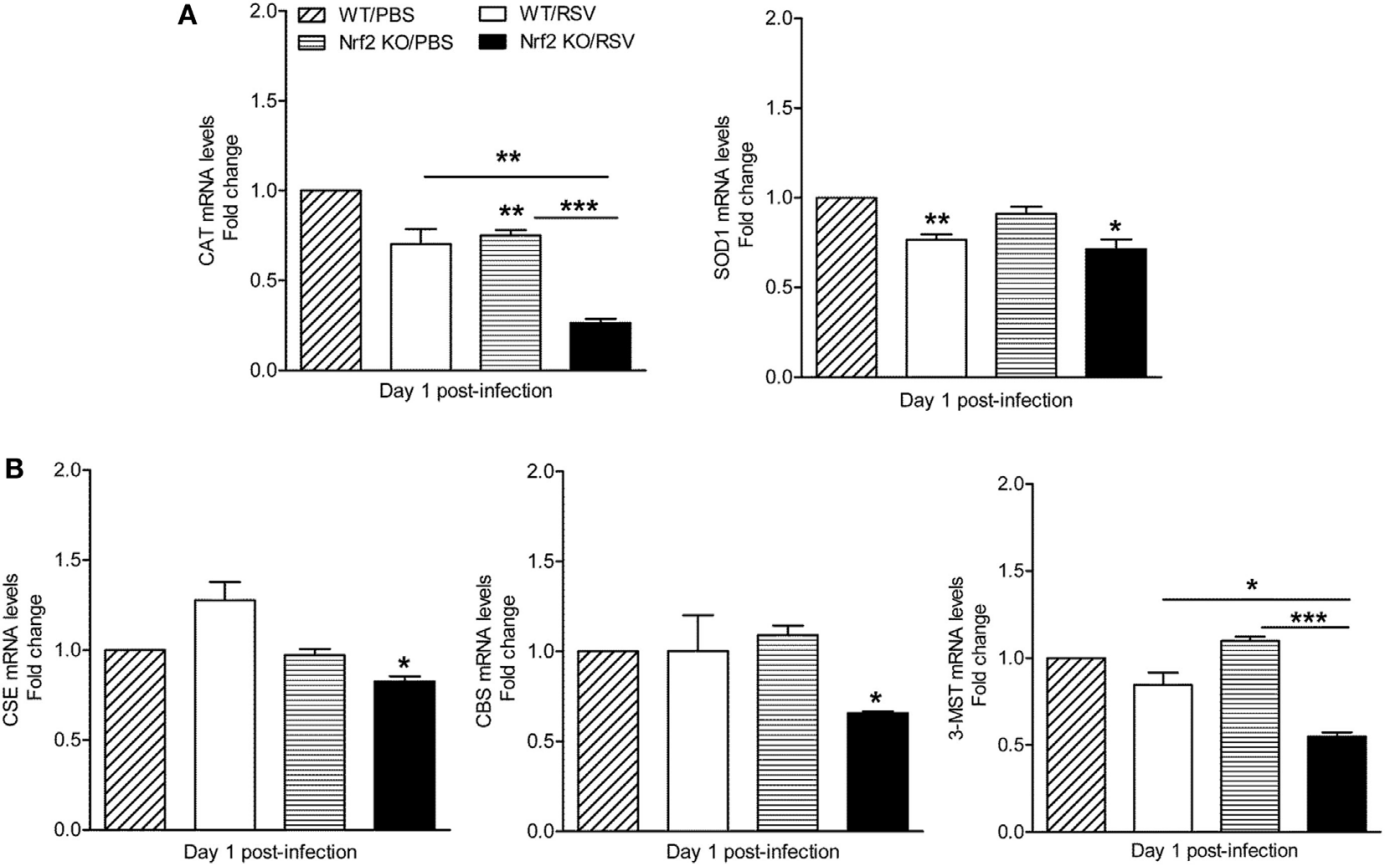
Decreased levels of antioxidant enzymes and hydrogen sulfide enzymes in NF-E2-related factor 2 (Nrf2)-deficient mice. Nrf2 KO and wild type (WT) mice were infected with respiratory syncytial virus (RSV) or PBS and harvested at day 1 post-infection to isolate total RNA from lungs. **(A)** Catalase (left panel) and Cu/Zn-superoxide dismutase-1 (right panel) gene expression were quantified by quantitative real-time PCR (and expressed as fold change). The bar graph represents mean ± SEM (*n* = 3 mice/group). *p < 0.05 Nrf2 KO/RSV vs. Nrf2 KO/PBS, **p < 0.01 Nrf2 KO/PBS vs. WT/PBS, WT/RSV vs. WT/PBS, and Nrf2 KO/RSV vs. WT/RSV, ***p < 0.001 Nrf2 KO/RSV vs. Nrf2 KO/PBS. **(B)** CSE, CBS, and 3-mercaptopyruvate sulfurtransferase mRNA levels were measured by quantitative real-time PCR (expressed as fold change). The bar graph represents mean ± SEM (*n* = 3 mice/group). *p < 0.05 Nrf2 KO/RSV vs. Nrf2 KO/PBS, and Nrf2 KO/RSV vs. WT/RSV, ***p < 0.001 Nrf2 KO/RSV vs. Nrf2 KO/PBS.

The Nrf2-ARE pathway regulates the expression of antioxidant and phase 2 metabolizing enzymes in response to oxidative stress (17) and RSV infection (16, 19). In addition, Nrf2 has been shown to regulate the expression of cystathionine γ-lyase (CSE) and cystathionine β-synthase (CBS) (35), two key enzymes that along with 3-MST are responsible for the synthesis of the endogenous gasotransmitter H_2_S (36). We have recently shown that endogenous H_2_S plays a critical antiviral function and mice that are genetically deficient in the CSE enzyme have increased RSV replication in the lung (24). Moreover, we have shown that RSV infection in human epithelial cells and in mouse lung is associated with decreased expression of AOE (16) and H_2_S-generating enzymes (24). Thus, we determined whether mice lacking Nrf2 had a relative deficiency in AOE and H_2_S-generating enzymes compared to WT controls following RSV infection. Lungs samples were harvested at day 1 post-infection to assess mRNA levels of catalase, Cu/Zn-superoxide dismutase (SOD1), CSE, CBS, and 3-MST. As shown in Figure 7A, lower levels of mRNA expression for catalase (left panel) and Cu, Zn-SOD1 (right panel) were observed in both RSV-infected Nrf2 KO and WT mice when compared with mock-inoculated mice. Noteworthy, mRNA levels for catalase, but not SOD1, were significantly lower in RSV-infected Nrf2 KO mice compared with WT (*p* < 0.01). In addition, mRNA levels of the H_2_S-producing enzymes CSE, CBS, and 3-MST were significantly decreased in Nrf2 KO mice when compared with WT after RSV infection (*p* < 0.05).

### Nrf2-Deficient Mice Have Greater Clinical Disease, Airway Obstruction, BAL Neutrophilia, and Cytokine Production Following Infection With hMPV

**Figure 8:**
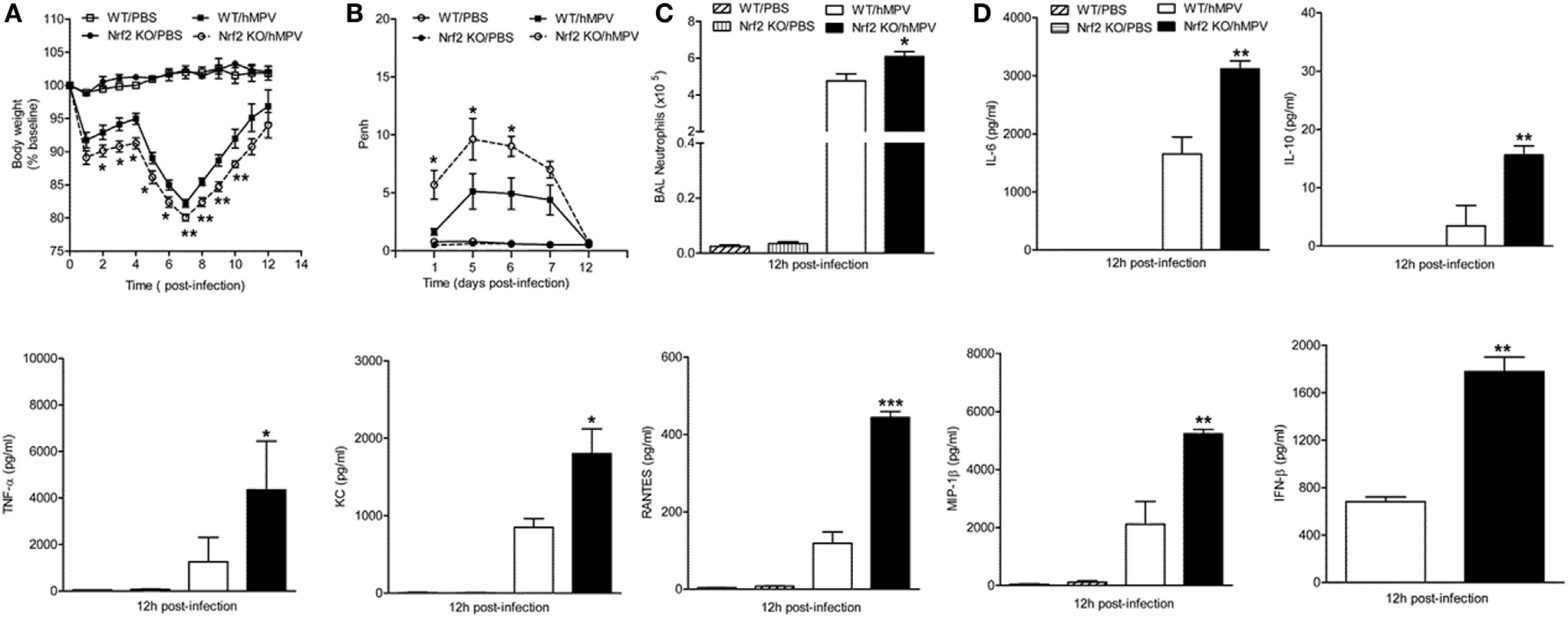
NF-E2-related factor 2 (Nrf2)-deficient mice have greater clinical disease, airway obstruction, bronchoalveolar lavage (BAL) neutrophilia, and cytokine production following infection with human metapneumovirus (hMPV). Nrf2 KO and Nrf2 WT mice were infected with hMPV or PBS. **(A)** Mice were monitored daily for changes in body weight. **(B)** Airway obstruction was assessed by unrestrained plethysmography as baseline Penh. **(C)** Total number of neutrophils was determined by cytospin analysis of BAL samples collected at 12 h p.i. **(D)** hMPV alters cytokine/chemokine production in Nrf2-deficient mice. BAL samples were collected at 12 h p.i. from each group of mice and assessed for cytokine/chemokine production by Bio-Plex and type I interferon by enzyme-linked immunosorbent assays. Data are expressed as mean ± SEM (*n* = 2–4 mice/group). *p < 0.05, **p < 0.01, ***p < 0.001 when compared with WT/hMPV.

Like RSV, hMPV is a member of the *Pneumoviridae* family and we have previously shown that it induces a progressive decrease of AOE expression levels in airway epithelial cells (AECs) and in the lung of infected mice (16, 22). We, therefore, tested the contribution of Nrf2 to hMPV-mediated disease, innate responses, and pulmonary function. For that, Nrf2 KO and WT mice were infected i.n. with hMPV (5 × 10^6^ PFU/mouse), monitored daily, pulmonary function measured, and BAL fluid analyzed at 12 h post-infection. As shown in Figure 8A and consistent with previous findings in experimental mouse models, hMPV-infected mice showed an initial phase of body weight loss, followed by a second phase, starting around day 5 post-infection. During both phases, hMPV-infected Nrf2 KO mice exhibited significantly more body weight loss and a delayed recovery, compared to hMPV-infected WT mice (*p* < 0.05 from days 2 to 5 p.i. and *p* < 0.01 from days 7 to 10 p.i.). Mock-inoculated mice from both groups did not play any significant weight loss over the 12 day monitoring period.

We have previously shown that hMPV infection in mice is also associated with airway obstruction and AHR, using both unrestrained whole-body plethysmography and invasive analysis of lung function (28). As shown in Figure 8B, hMPV-infected Nrf2 KO mice showed consistently increased baseline obstruction compared to WT-infected mice (*p* < 0.05), starting at day 1 post-infection, peaking around day 5, and returning to baseline by day 12 post-infection. They also exhibited a significant dose-dependent increase in AHR in response to aerosolized methacholine at day 1 post-infection, compared to WT mice, with no differences between hMPV-infected groups by day 12 post-infection (data not shown).

Airway neutrophilia is characteristic of experimental hMPV infection, including adult (28) and older mice (37). In this study, BAL samples were collected 12 h after inoculating mice with hMPV or PBS, and differential cell count was performed. We focused on the neutrophil recruitment at earlier time points post-infection based on previous observations that indicate that neutrophil recruitment peaks by day 1 after hMPV infection. As shown in Figure 8C, BAL neutrophil counts were greater in hMPV-infected Nrf2 KO compared WT mice (*p* < 0.05).

To further elucidate the role Nrf2 in hMPV infection, we determined the profile and concentration of cytokine and type I IFN levels and compare it to that of WT mice. Cell-free supernatants from BAL samples were analyzed by a Bio-Plex array, and tested for IFN-β production by ELISA. As shown in Figure 8D, levels of IL-6 (*p* < 0.01), IL-10 (*p* < 0.01), TNF-α (*p* < 0.05), CXCL1 (*p* < 0.05), CCL2 (*p* < 0.05), CCL5 (*p* < 0.001), and CCL4 (*p* < 0.01) were significantly increased in hMPV-infected Nrf2 KO compared with WT mice. No significant difference was observed in the production of other cytokines and chemokines in the array. Overall, similar to what we observed with RSV our results demonstrate that hMPV infection in Nrf2 KO mice causes increased body weight loss, airway obstruction, neutrophilic inflammation, and increased levels of proinflammatory cytokines.

## Discussion

The results of this study show that mice genetically deficient in Nrf2 developed enhanced clinical disease, airway inflammation and pathology, and significantly greater lung viral titers following experimental infection with RSV. In particular, we have shown that compared to control mice, Nrf2 KO mice lost more body weight and had increased airway obstruction at day 1 post-infection (Figure 1), a time point which was characterized by a remarkable increase in airway neutrophilia (Figure 4). As mentioned in the introduction, neutrophil migration to the airways is a feature of naturally acquired RSV infections in human, is considered a hallmark of bronchiolitis and is recapitulated by BAL neutrophilia in the murine model of experimental infection (38). These features of RSV infections were associated with an increased innate and inflammatory cytokines and chemokines, and overall reduced expression of the antioxidant machinery, compared to control WT mice (Figures 5 and 7). These findings are in agreement with our previous observations in Nrf2-competent BALB/c mice, in which RSV infection leads to a significant decrease in the expression of most AOE involved in maintaining cellular oxidant–antioxidant balance in the lung. This process is associated with a progressive decrease in nuclear protein levels of Nrf2 (16), *via* increased Nrf2 ubiquitination and its degradation through a proteasomal pathway, which we observed both *in vitro* epithelial cells and in RSV-infected mouse lung (39). Early innate/inflammatory events in the lung appeared to be critical in shaping the overall antiviral response and some of the disease features and as evident in our studies by the observation that peak lung viral titers at day 5 were higher in Nrf2 KO mice and lung histopathology at day 7 was characterized by significantly more alveolitis and interstitial pneumonitis compared to control Nrf2-competent mice. However, in agreement with another report (19), we did not observe sustained airway obstruction or AHR to methacholine challenge at the later time point tested.

The mechanisms underlying the increased RSV replication/gene copies in the lung of Nrf2 KO mice are unclear. Similar to our findings, adult Nrf2 KO mice on ICR background were shown to have increased peak viral replication and shedding (19). The relative defect in the antioxidant defense system and enhanced oxidative response in absence of Nrf2 (16, 19) could in part explain this finding. In previous work, treatment of AECs with the salen–manganese complexes EUK-8 or EUK-189, which possess superoxide dismutase, catalase, and glutathione peroxidase activity, strongly reduced RSV-induced ROS formation by increasing cellular AOE enzymatic activity and reduced viral replication when used at higher doses (40). In other models of experimental viral infections (influenza), exogenous treatment with the AOE catalase significantly reduced viral titers in the lung of mice (41). Nrf2 has also been shown to affect Th balance, i.e., favoring Th1 responses, while oxidative stress might be involved in the loss of naïve T cells and decrease in Th1 immunity (42). As such, changes in innate immunity, including macrophage and dendritic cell antigen presenting functions, which occur in mice deficient in Nrf2 and consequent increased oxidative stress has been shown to shift the Th1/Th2 balance (43). Although we have not conducted extensive work to address the robustness and nature of T cells responses in RSV-infected Nrf2 KO mice and potential involvement in the reduced antiviral response, we observed greater concentrations of IL-13 and IL-10 in BAL of RSV-infected Nrf2 KO at day 1 post-infection, while on the other hand levels of IFN-γ were greater in Nrf2 KO at days 5 and 7 compared to WT controls (Figure 5). Thus, cytokine levels in the BAL fluid suggest a skewed Th2 response only in the initial phase of infection. Choi et al. have reported a more prolonged increased of IL-13 and IL-10 in Nrf2 compared to WT mice (up to day 7 post-infection), which could explain the presence of BAL eosinophils in their study (rather than neutrophils as in our study). We suspect that the mouse background of our study (C57BL/6) and the previous study (ICR) (19), as well as the strain (long vs. A2) and dose of RSV infection (smaller dose in Choi’s study) may be the reason for these contrasting findings.

Another potential explanation for the increased RSV replication is the relationship between Nrf2 and the H_2_S-generating enzymes, as suggested by the significant reduction in expression of CSE, CBS, and 3-MST which we observed in RSV-infected Nrf2 KO mice (Figure 7). We have recently described antiviral properties of the cellular endogenous H_2_S pathway. In particular, we have shown that inhibition of CSE, a key enzyme in the biosynthesis process of H_2_S, is associated with enhanced RSV replication in human epithelial cells (26) and mice genetically deficient in CSE showed ~50% increase in RSV peak lung titer. Moreover, treatment of RSV-infected BALB/c mice with H_2_S pharmacologic donors significantly reduced viral replication, indicating that endogenous H_2_S plays an important role in controlling RSV replication (24). The involvement of the H_2_S pathway in the observed increase in RSV replication in Nrf2 KO mice was further suggested by the significant reduction in lung titers when Nrf2 KO mice were treated with the H_2_S donor GYY4137 (Figure S1 in Supplementary Material). Not surprisingly, based on the complex nature of interaction between RSV and type I IFNs, the increase in peak RSV replication in the lung of mice lacking Nrf2 occurred despite increased levels of secreted type I IFN compared to Nrf2-competent animals (Figure 5C).

Our results showed that levels of several innate cytokines and chemokines in BAL were significantly increased in absence of Nrf2 gene, suggesting that it plays a central role in modulating/inhibiting excessive viral-mediated production of pro-inflammatory mediators. There may be multiple mechanisms to explain the inhibitory activity of Nrf2, one being its inhibitory activity on the NF-κB transcription factor, which is the major arm of the innate immune response that controls the RSV-induced gene program in the lung. Indeed, our results in RSV-infected mice (Figure 6) and previous work in model of experimental sepsis (43) demonstrate greater nuclear translocation of RelA in the lung in absence of Nrf2. Although is not clear how Nrf2 interferes with RSV signaling to inhibit NF-κB, several lines of evidence, including our own work in epithelial cells and in mice indicate that the redox status of the cells modulates RSV-induced NF-κB activation (31, 44). Thus, overall reduction in the Nrf2-dependent cellular antioxidant system, which occurs in RSV infection (15, 16) and in Nrf2-deficient mice (19) and (Figure 7), would result in an unbalanced production of ROS in the airway mucosa (12) and contribute to the activation of NF-κB and other transcription factors, including STAT, and IRF proteins that control the innate/inflammatory gene network (13, 14). Other mechanisms underlying Nrf2-mediated anti-inflammatory properties in RSV infection could include its suppressive activity on macrophage cytokines. In this regard, alveolar macrophages (AMs) significantly contribute to the production of key innate and inflammatory cytokines in the lung in response to RSV, including type I IFN, IL-6, and TNF-α, as we showed in studies in which these cells were depleted prior to viral inoculation (25). Nrf2 has been shown to bind to the proximity of cytokine genes (including IL-6 and IL-1β) in macrophages and to inhibit RNA Pol II recruitment, a process independent of the Nrf2-binding to motif and ROS levels (45).

We have previously shown that similar to RSV, hMPV induces a progressive decrease in AOE expression levels in AECs (22) and in the lung of infected mice (16). As member of the *Pneumoviridae* family, hMPV was first isolated in 2001 and since been characterized as an important cause of acute respiratory tract infections in the young (46, 47). Recent prospective surveillance studies conducted in the US over 3–6 seasons have shown that the annual rate of hMPV-associated hospitalization in children is the same as the hospitalization rate associated with influenza virus (48). The pathophysiology of hMPV infection is largely unknown, and some of the scientific information has been extrapolated from the RSV research literature, rather than by direct or comparative side-by-side studies. Herein, we show for the first time that lack of Nrf2 in hMPV-infected adult mice is associated with increased secretion of cytokines in BAL, airway neutrophils, exacerbated body weight loss, and sustained airway obstruction (for more than a week), compared to control Nrf2-competent mice. Therefore, it appears that experimental infections with RSV and hMPV cause a spectrum of innate and inflammatory responses that are similarly affected by Nrf2-dependent pathways, although some of the evidence of oxidative-mediated injury that we have reported in human natural RSV infections has yet to be investigated in hMPV infections (16).

In conclusion, these results suggest that Nrf2 is a critical regulator of innate, inflammatory, and disease-associated responses in the airways of mice infected with viruses that are members of the *Paramyxoviridae* family. Importantly, the results of this study suggest that Nrf2-dependent genes, including those controlling the cellular antioxidant and H_2_S-generating enzymes and cytokines can affect several aspects of the antiviral response, such as airway neutrophilia, clinical disease, airway obstruction, and viral replication. Translating mouse experimental models into human disease, this study and previous ones by us and others suggest a potential new mechanism of RSV pathogenesis driven by viral-mediated oxidative stress damage of the airways which is not balanced by an adequate AOE response due to direct degradation of Nrf2 by RSV and/or by functional deficits in Nrf2 and AOE gene expression, which are genetically determined or linked to age-dependent maturation (16, 19). In our previous study of nasopharyngeal secretions obtained from a relatively small population of RSV-infected children with different spectrum of airway disease severity, we found a significant increase in markers of oxidative injury and a significant decrease in AOE expression, which correlated with the severity of lung disease (16). The known developmental process of the AOE system and of the H_2_S-generating enzymes that starts during fetal life and is characterized by a certain degree of immaturity in the neonatal period and early infancy may contribute to the severity of RSV infections that occur during this vulnerable period of life (49, 50). Therefore, these findings may have implications for the development of therapeutic interventions aimed to increase the antioxidant tone or control the redox state in the airways at the time of RSV infections, including measures to acutely increase Nrf2 activity. These approaches have shown significant benefits *in vitro* and in experimental animal models of RSV infections by modulating cytokine secretion, reducing lung inflammation, airway dysfunction, and viral replication (12, 19, 40).

## Ethics Statement

All procedures involving mice in this study were performed in accordance with the recommendations in the Guide for the Care and Use of Laboratory Animals of the National Institutes of Health. The protocol was approved by the Institutional Animal Care and Use Committee of the University of Texas Medical Branch at Galveston. The study has been approved by the UTMB IACUC (protocol number 9001002 and 0808049).

## Author Contributions

TI contributed to the design of the experiments, performed the experiments presented in this manuscript, and wrote the manuscript. RG provided the overall conceptual design of these studies, reviewed and edited the final version of the manuscript. AC contributed to the design of the study and supervised the experiments related to NF-κB assays. ES performed the pathology analysis of lungs. All authors read and approved the manuscript.

## Conflict of Interest Statement

The authors declare that the research was conducted in the absence of any commercial or financial relationships that could be construed as a potential conflict of interest.

## Supplementary Material

Figure S1 GYY4137 treatment reduces respiratory syncytial virus (RSV) replication in NF-E2-related factor 2 (Nrf2) KO mice. Nrf2 KO and wild type (WT) mice were treated intranasally with GYY4137 (50 mg/kg body weight) or an appropriate volume of vehicle (PBS) 1 h before, 6 and 20 h after infection. Mice were inoculated with either RSV (1 × 10^7^ plaque-forming units/mouse) or PBS. At day 5 after infection, lungs were excised and viral replication was determined by plaque assay. Data are expressed as mean ± SEM (*n* = 3 mice/group). **p* < 0.05 Nrf2 KO/PBS/RSV compared with WT/PBS/RSV, Nrf2 KO/GYY4137/RSV compared with WT/GYY4137/RSV, ****p* < 0.001 WT/PBS/RSV compared with WT/GYY4137/RSV, and Nrf2 KO/PBS/RSV compared with Nrf2 KO/GYY4137/RSV.
