# Supplementary figures and images for: Protective Role of Nuclear Factor Erythroid 2-Related Factor 2 Against Respiratory Syncytial Virus and Human Metapneumovirus Infections
Source: Front Immunol. 2018 Apr 23;9:854. doi: 10.3389/fimmu.2018.00854 (PMC5925606; doi:10.3389/fimmu.2018.00854)

Figure S1

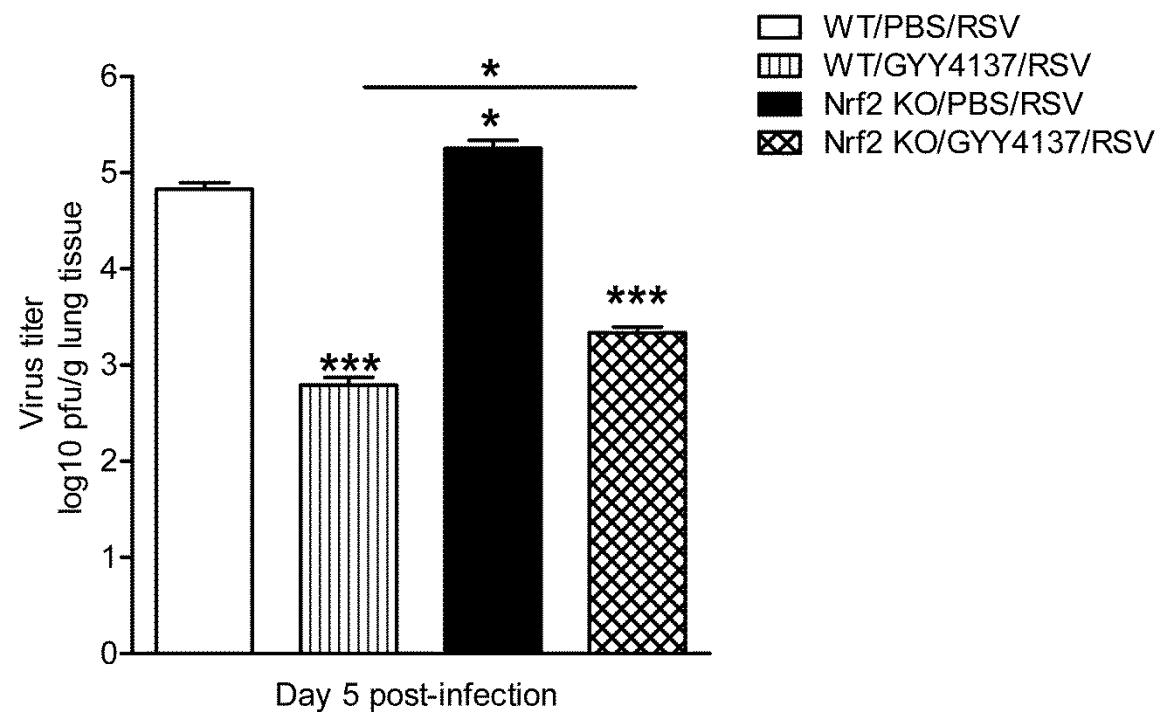

Supplement: Figure S1 — GYY4137 treatment reduces respiratory syncytial virus (RSV) replication in NF-E2-related factor 2 (Nrf2) KO mice. Nrf2 KO and wild type (WT) mice were treated intranasally with GYY4137 (50 mg/kg body weight) or an appropriate volume of vehicle (PBS) 1 h before, 6 and 20 h after infection. Mice were inoculated with either RSV (1 × 10^7^ plaque-forming units/mouse) or PBS. At day 5 after infection, lungs were excised and viral replication was determined by plaque assay. Data are expressed as mean ± SEM (n = 3 mice/group). *p < 0.05 Nrf2 KO/PBS/RSV compared with WT/PBS/RSV, Nrf2 KO/GYY4137/RSV compared with WT/GYY4137/RSV, ***p < 0.001 WT/PBS/RSV compared with WT/GYY4137/RSV, and Nrf2 KO/PBS/RSV compared with Nrf2 KO/GYY4137/RSV. [file image_1.PDF]
